# 25-hydroxy Vitamin D Levels in Pediatric Asthma Patients and its Link with Asthma Severity

**DOI:** 10.7759/cureus.4302

**Published:** 2019-03-22

**Authors:** Gulten Ozturk Thomas, Engin Tutar, Gulnur Tokuc, Sedat Oktem

**Affiliations:** 1 Pediatrics, Dr. Lutfi Kirdar Training & Research Hospital, Istanbul, TUR

**Keywords:** asthma, 25-hydroxy vitamin d, disease severity

## Abstract

Aim

Although the relationship between 25-hydroxy (25-OH) vitamin D and asthma is known, it is unknown if 25-OH vitamin D levels are correlated with asthma severity in pediatric patients. The aim of this study was to compare the blood 25-OH vitamin D levels of asthma patients and healthy control groups and to evaluate any correlation between asthma severity and blood 25-OH vitamin D levels in pediatric asthma patients.

Methods

This is a cross-sectional study which shows the 25-OH vitamin D levels of asthma patients and compared to healthy controls followed by a tertiary pediatric clinic. We investigated the effect of 25-OH vitamin D levels on the severity of asthma. The severity of asthma was determined mainly by the duration of asthma diagnosis, a number of attacks in the previous year, anti-inflammatory medication usage in a previous year, atopy presence in the family, skin prick test positivity, and immunoglobulin E (IgE) levels of asthma patients.

Results

Compared with control groups, asthma patients had significantly lower 25-OH vitamin D, calcium (Ca) levels, and higher number of patients who had a 25-OH vitamin D deficiency, (p<0.0001, p<0.0001, p<0.0001, respectively). We found a correlation between blood 25-OH vitamin D levels and force expiratory capacity in one second (FEV1) and forced vital capacity (FVC) respectively (p< 0.001, r=0.512), (p< 0.001, r=0.513). There was an association between FEV1, FVC and blood 25-OH vitamin D levels in terms of deficient levels (<20 ng/mL) or insufficient levels (≥20 and <30 ng/mL) (p<0.001, r=0.459), (p< 0.001, r=0.450), respectively.

Conclusions

The 25-OH vitamin D levels were lower in pediatric asthma patients with worse spirometry results. Effects of Vitamin D supplementation need to be evaluated by well-designed studies.

## Introduction

Asthma - a chronic inflammatory disease characterized by recurrent episodes of wheezing, shortness of breath and coughing due to hyper-responsiveness of the airways, is one of the most common chronic diseases seen in childhood [1-2]. The prevalence of both asthma and 25-hydroxy (25-OH) vitamin D deficiency is reported to have increased in children [3-4] and one hypothesis for the rising prevalence of asthma involves 25-OH vitamin D deficiency [5-6]. Information from the International Study of Asthma and Allergy in Children (ISAAC) study group shows the highest prevalence of asthma in well-developed western countries [7]. Some authors have argued that 25-OH vitamin D has more of a deleterious effect on allergic pathogenesis [8]. Epidemiologic studies suggest that 25-OH vitamin D deficiency is also associated with an increased incidence of asthma symptoms [5]. There is limited data on 25-OH vitamin D levels in children with asthma, as well as on what features of pediatric asthma are associated with the 25-OH vitamin D levels. The prevalence of 25-OH vitamin D insufficiency/deficiency is higher for children living in Turkey [9]. A recent study on children with asthma from Costa Rica showed a significant inverse association between the 25-OH vitamin D levels and use of anti-inflammatory medication (either inhaled corticosteroid or leukotriene inhibitor) in the previous year, total immunoglobulin E (IgE), and eosinophil count [10]. These important findings require confirmation. The present knowledge about the relationship between 25-OH vitamin D and asthma is still not enough to consider 25-OH vitamin D as an alternative or adjuvant treatment option in childhood asthma.

The aim of our study is to investigate blood 25-OH vitamin D levels of asthma patients followed by our outpatient clinic, compare it with healthy control groups and to evaluate any correlation between asthma severity and the blood 25-OH vitamin D levels.

## Materials and methods

Subjects

Children aged between 6 and 15 years with a diagnosis of asthma followed by the Pediatric Pulmonology Clinic of Dr. Lutfi Kırdar Kartal Research and Training Hospital were included in the study. A control group of healthy patients in the same age range was selected from among patients applying for a routine check-up to general pediatrics outpatient clinic. Patients were excluded if history revealed or it was documented in patients' files that they were taking 25-OH vitamin D supplements, if they had additional chronic pulmonary conditions or if any infectious or chronic diseases were present. Data were collected between September - December 2010 and September - December 2011. The study was designed as such to exclude any bias originating from the effect of seasonal sunshine on the blood 25-OH vitamin D levels. Patients and controls were enrolled and their data were collected at the same season/time frame.

A questionnaire collecting sociodemographic information with questions to determine asthma severity in the patients was prepared by the authors and completed by patients’ parents. 25-OH vitamin D supplementation history in the first year of life was queried.

The protocol was approved by the local Ethics Committee and informed consents were obtained from each parent, including consent for publication of data.

Biochemical analysis

Serum 25-OH vitamin D levels were analyzed photometrically by ROCHE DPP Modular system (Roche, Germany). Calcium (Ca), phosphorus (P) and alkaline phosphatase (ALP) levels were checked to exclude the patients with laboratory findings of rachitism. We categorized 25-OH vitamin D levels as deficient (<20 ng/mL), insufficient (≥20 and <30 ng/mL), and sufficient (≥30 ng/mL) [11]. For quality control, samples were run in duplicate and the average measurement was taken.

Pulmonary function tests

Vital capacity and flow rates were measured by spirometry in accordance with the criteria of the American Thoracic Society (ATS) while patients were awake and in the seated position [12]. Spirometry was performed by one of the authors. The values of spirometry were expressed as the ratio of percentage of normal values based on age, gender, and height. The best value of a minimum of the three adequate measurements was taken. Reference values obtained in the study by Knudson, et al. [13] were used.

Statistical analysis

Statistical analysis was performed by Statistical Package for the Social Sciences (SPSS) 16.0. Continuous variables were described through means and standard deviations, whereas categorical variables were presented as proportions. Categorical variables were compared with Chi-square and with Fisher’s exact test when 20% of the expected frequencies were less than five. Continuous variables among two groups were compared with Student T-test, since the data followed a normal distribution. Bivariate correlations were evaluated through Pearson correlation. Spearman correlation was used to analyze the relation between FEV1, FVC and blood 25-OH vitamin D levels in terms of deficient or insufficient. A p-value < 0.05 was considered as statistically significant.

## Results

Subject characteristics

Seventy-three patients with asthma (40 male, 33 female) and an average age of 10.2±2.7 years (range 6-15 years) were enrolled in the study. The control group consisted of 44 healthy subjects (24 males, 20 females) without any chronic disease or infection, in the same age range (average age: 10.0±2.6 years). There was no statistically significant difference between the two groups in terms of age, gender, P and ALP levels. There was no statistical difference between the 25-OH vitamin D levels according to gender and age (p>0.05).

Demographic information, biochemical analyses and 25-OH vitamin D data for all subjects are shown in Table 1.

**Table 1:** Demographic, biochemical analyses and 25-hydroxy vitamin D level data for asthma patients and control subjects #: Student t test, +: Chi square test, Ca: Calcium, P: Phosphorus, ALP: Alkaline phosphatase, 25-OH: 25-hydroxy

	Asthma Group	Control Group	p
Age (Years)	10.2±2.7	9.95±2.56	p=0.636
Gender , Male/Female (n)	40/33	24/20	P=0.979
Average 25-OH vitamin D level, ng/ml	12.1±7.43	19.8±1.02	p<0.001
Patients with 25-OH vitamin D deficiency (<20 ng/ml) - n (%)	60 (82.2)	21(47.7)	p<0.001
Patients with 25-OH vitamin D insufficient (≥20 and <30 ng/mL) - n (%)	13(17.8)	15(34)	
Patients with 25-OH vitamin D sufficient (≥30 ng/mL) - n (%)	0	8(18.2)	
Ca, mg/dl	9.47±0.4	9.82±0.52	P<0.001
P, mg/dl	4.62±0.54	4.72±0.53	p=0.196
ALP, mg/dl	210±82	226.35±72	p=0.372

Compared with control groups, asthma patients had significantly lower 25-OH vitamin D and Ca levels, and the asthma group had higher number of patients who had 25-OH vitamin D deficiency (p<0.001, p<0.001, p<0.001, respectively).

Clinical associations

There was no relationship between the blood 25-OH vitamin D levels and the time since asthma diagnosis, the number of attacks, inhaled steroid, systemic steroid or anti-inflammatory (either inhaled corticosteroşds or leukotriene inhibitors) medication use during the last year, atopy in the family, skin prick test positivity, 25-OH vitamin D usage in first year of life and Ig E levels in asthma patients (p>0.05).

**Figure 1:**
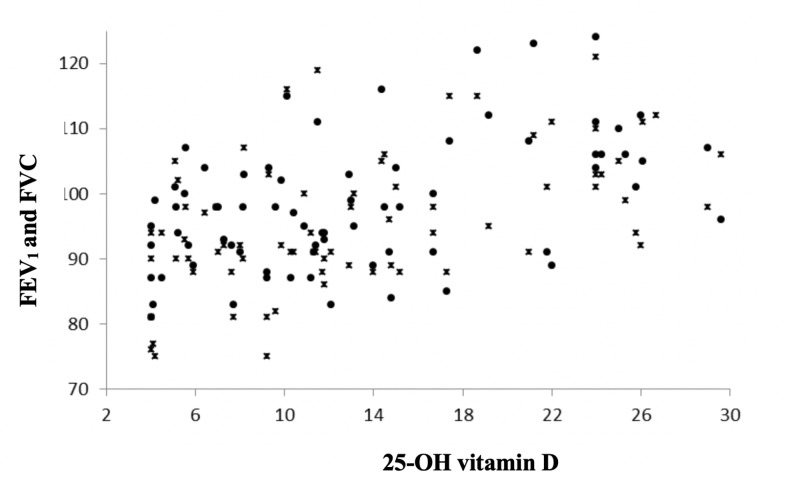
Association between blood 25-hydroxy (25-OH) vitamin D levels and pulmonary function tests in asthma patients x: Forced expiratory volume in the first second, circle: Forced vital capacity

The blood 25-OH vitamin D levels were found to correlate with forced expiratory volume in the first second (FEV1) and forced vital capacity (FVC) (p< 0.001, r=0.512 and p< 0.001, r=0.513, respectively.) Also, there was an association between FEV1, FVC and blood 25-OH vitamin D levels for the groups with deficient levels (<20 ng/mL) or insufficient levels (≥20 and <30 ng/mL) (p< 0.001, r=0.459 and p< 0.001, r=0.450 respectively.) The relationship between FEV1, FVC and blood 25-OH vitamin D levels are shown in Figure 1.

There was no statistically significant difference between 25-OH vitamin D levels and inhaled steroid usage in last 1 year (9.15±5.18 ng/ml vs 9.65±4.78 ng/ml, p=0,672). There was no correlation between the IgE levels of the patients and 25-OH vitamin D levels (p=0.677, r=-0.50). Also, there was no association between IgE and blood 25-OH vitamin D levels for patients with levels lower than 20 ng/ml or between 20-30 ng/ml (p>0.86, r=-20).

## Discussion

We found a remarkably high prevalence of low 25-OH vitamin D levels among the 6-15 year old control group and the asthma group. This finding clearly indicates that 25-OH vitamin D deficiency is an important health problem in children living in Istanbul, Turkey. The insufficiency levels may be caused by limited sunshine exposure due to individuals’ spending more time indoors or avoiding sunshine intentionally for fear of air pollution, skin pigmentation etc, or due to the covering of skin and low 25-OH vitamin D intake [14]. Also 25-OH vitamin D receptor (VDR) gene polymorphisms might be an important factor for genetic susceptibility to 25-OH vitamin D deficiency in the Turkish population [15].

The functional consequences of VDR polymorphism in the population and its correlation with 25-OH vitamin D levels is an interesting topic for research. VDR polymorphism may explain why some patients with extremely low 25-OH vitamin D levels are asymptomatic, and why different studies regarding vitamin D levels and asthma severity have found different results [16-17] however we could not measure 1.25 dihysroxyvitamin D levels in our study. This needs to be further studied.

Another factor to be pointed out is that the usage of food fortified by 25-OH vitamin D is extremely low in Turkey when compared to other countries. In a study, 25-OH vitamin D deficiency was more prevalent among infants who did not receive supplementation with 25-OH vitamin D, and the seasonal effects were more pronounced in this group (78% were deficient in winter vs 4% in summer) [16]. It should be noted, however, that one weak point of our study was that we did not determine 25-OH vitamin D levels during the summer.

Another weak point of our study is the fact that we did not have the chance to evaluate other possible factors like race, sun exposure time and gene polymorphisms which might have important effects on the blood vitamin D levels. The fact that asthma patients might go outdoors less often compared to healthy children might be another reason for low vitamin D levels.

There was correlation between the 25-OH vitamin D levels and FEV1, FVC values as noted by other studies which include the observations that 25-OH vitamin D levels are inversely associated with degree of worsening airflow limitation among asthmatics [17]. There was no relation between blood 25-OH vitamin D levels and the time from asthma diagnosis; number of attacks, inhaled steroid usage, anti-inflammatory medication (either ICS or leukotriene inhibitor) usage or systemic steroid usage in the previous year; atopy in the family, skin prick test positivity, 25-OH vitamin D usage in first year of life and the IgE level in asthma patients. Searing et al. showed that the 25-OH vitamin D levels were markedly low in asthma patients using oral or inhaler steroids to control the disease and also patients with low 25-OH vitamin D levels had more frequent attacks and were in more steroid need compared to patients with normal 25-OH vitamin D levels [18].

Some studies showed that 25-OH vitamin D supplementation might increase the effect of glucocorticoids in steroid resistant patients by increasing IL-10 production which is an anti-inflammatory mediator affecting the peripheral mononuclear cells [11,19-20]. One recent study showed that vitamin D alleviates airway remodeling by down-regulating the activity of Wnt/β-catenin signaling pathway [21].

Our study population were found to have a mild asthma severity (mean FEV1= 96.1±11.9, mean FEV1/FVC=95.5±9.4). A majority of the patients (n=61, 83%) had no symptoms at the time of the study. There may be two reasons for our findings of low 25-OH vitamin D levels not being consistency with the severity of the disease. First, our patients had mild asthma with few attacks per year, and all of our population had 25-OH vitamin D levels below normal range. Secondly, genetic predisposition may influence the manifestation of 25-OH vitamin D deficiency. Although a large number of breast-fed infants have low serum 25-OH vitamin D levels, only a few of them manifest overt symptoms of 25-OH vitamin D deficiency. Moreover, obvious environmental factors such as lack of exposure to sunshine or inadequate 25-OH vitamin D intake are not associated with 25-OH vitamin D deficiency in some patients. A study speculated that genetic predisposition may influence the manifestation of 25-OH vitamin D deficiency [22].

In some studies it was emphasized that 25-OH vitamin D supplementation in the first year of life has a negative correlation with pulmonary symptoms later in life whereas others pointed out that 25-OH vitamin D supplementation in the first year of life might increase the likelihood of asthma in the third decade of life [5,23]. We did not find any correlation between 25-OH vitamin D supplementation in the first year of life and disease severity in our study. In a very recent study perinatal Vitamin D supplementation attenuates asthma development following traffic-related particulate matter exposure but does not help attenuate symptoms after asthma has been developed [24].

We found the 25-OH vitamin D levels of asthma groups lower than control groups. This supports the findings of other studies. Gupta et al. published a paper in 2011 showing that low 25-OH vitamin D levels increases the likelihood of asthma and other chronic pulmonary diseases by increasing airway smooth muscle mass [25].

Despite the great number of recently published studies on the relation of vitamin D with asthma there is still no consensus on considering 25-OH vitamin D as an alternative single treatment for inflammatory diseases but is only adviced as a supplementation [26-27].

## Conclusions

In this study, we found lower serum 25-OH vitamin D levels in asthmatic children when compared with controls, and we also found a relationship between serum 25-OH vitamin D levels and FEV1, FVC. We were able to show that 25-OH vitamin D insufficiency in childhood asthma is common yet similar to the general population. We were unable to find a relationship between the serum 25-OH vitamin D levels and several markers of allergy and asthma severity. Our data suggest that additional prospective research needs to be performed to determine the potential beneficial role that 25-OH vitamin D has on asthma.
